# A Case of Plateau Iris Syndrome: A Diagnosis and Management Dilemma

**DOI:** 10.7759/cureus.105436

**Published:** 2026-03-18

**Authors:** Nur Izzah Husna Saaid Zaidun, Maisyatun Nazihah Mohamad Nawi, Rohana Abdul Rashid, Khairidzan Mohd Kamal, Qi Zhe Ngoo

**Affiliations:** 1 Ophthalmology, International Islamic University Malaysia (IIUM), Kuantan, MYS; 2 Ophthalmology, Universiti Sains Malaysia, Kota Bharu, MYS; 3 Ophthalmology, Hospital Tengku Ampuan Afzan, Kuantan, MYS

**Keywords:** cataract extraction, peripheral iridoplasty, plateau iris syndrome, primary angle-closure glaucoma (pacg), ultrasound biomicroscopy

## Abstract

This is a case report of primary angle-closure glaucoma (PACG) with persistent high intraocular pressure (IOP) despite patent laser peripheral iridotomy (LPI) and treated with argon laser peripheral iridoplasty (ALPI) possibly due to plateau iris syndrome. A 49-year-old gentleman with underlying hypertension, subclinical hypothyroidism, seizure, and ocular history of bilateral normotensive glaucoma (NTG) on topical latanoprost 0.005% every night (ON) presented with acute onset of left eye pain and redness. His IOP was found to be high with shallow anterior chamber depth and the presence of mild cataract. He was initially diagnosed with bilateral eye PACG and started with four topical antiglaucoma medications; subsequently, LPI was commenced. At subsequent follow-up, the patient reported persistent occipital headache. The IOP remained high despite compliance with all topical medications and having the enlarged patent PI over both eyes. Ultrasound biomicroscopy (UBM) was done and showed the possibility of plateau iris syndrome. He was treated with ALPI, and the IOP was reducing in trend; however, it still did not reach the optimum level. He subsequently underwent left eye phacoemulsification with intraocular lens (IOL) implantation. IOP of both eyes subsequently was able to reach the optimum level with a reduction in the number of antiglaucoma eyedrops used.

Managing plateau iris syndrome may require a combination of therapies to effectively lower the IOP. ALPI may not solely be as effective as its combination with cataract removal surgery in managing plateau iris syndrome.

## Introduction

Glaucoma is a heterogeneous group of optic neuropathies characterized by progressive damage to the optic nerve, resulting in a distinctive pattern of retinal ganglion cell loss and irreversible visual field deficits, which can ultimately lead to blindness [1]. It is the world's leading cause of irreversible blindness. Glaucoma is broadly categorized into primary and secondary forms and can be further subdivided by the morphology of the anterior chamber angle into open-angle and angle-closure variants [2]. Geographically, the prevalence of primary open-angle glaucoma (POAG) is highest in Africa at 4.20%, while primary angle-closure glaucoma (PACG) is most frequent in Asia at 1.09% [3]. Global data for the adult population aged 40-80 reflects a significant upward trend in the disease burden: the number of affected individuals rose from 64.3 million in 2013 to 76 million in 2020 and is projected to reach 111.8 million by 2040 [3]. PACG is distinguished by physical contact between the peripheral iris and the trabecular meshwork, which impedes the outflow of aqueous humor (AH) and leads to an increase in intraocular pressure (IOP) [2]. Hence, this can manifest acutely as an ocular emergency which may lead to rapid and severe vision loss due to ischemic damage to the optic nerve [2]. To manage acute pupillary block in PACG, laser peripheral iridotomy (LPI) is typically employed to create a fluid bypass [1]. While LPI effectively resolves pupillary block, a subset of patients with PACG may continue to experience angle closure due to non-pupillary block mechanisms, a condition referred to as plateau iris syndrome (PIS) or plateau iris configuration [4,5]. 

PIS is anatomically characterized by an anteriorly positioned or abnormally large ciliary body that pushes the iris root forward, crowding the anterior chamber angle despite a flat peripheral iris contour, often described as a "double-hump sign" on gonioscopy [1,6]. This physical configuration results in persistent iridotrabecular contact (ITC) even after a patent iridotomy has eliminated pupillary block [5]. Therefore, early diagnosis and management of plateau iris are extremely important as the patient may develop chronic or recurrent angle-closure attacks and sustained elevation of IOP, directly contributing to PACG [5]. Diagnostic advancement, such as ultrasound biomicroscopy (UBM), is utilized to visualize the ciliary body's anomalous anterior rotation and loss of ciliary sulcus space, which are not discernible with standard anterior segment optical coherence tomography (AS-OCT) [5]. Clinically, PIS is often suspected in younger patients (typically 30-50 years old, predominantly female) who present with persistently narrow angles or develop recurrent angle-closure attacks despite a patent laser iridotomy [7,8]. Management strategies for PIS may involve argon laser peripheral iridoplasty (ALPI) to flatten the peripheral iris or medical therapies like low-dose pilocarpine to constrict the pupil and pull the iris away from the trabecular meshwork, aiming to prevent further angle closure and subsequent glaucomatous damage [5]. However, the effects of ALPI are frequently transient [9]. In cases of medical therapy and ALPI failing to control glaucoma in PIS patients, surgical treatment may be considered. Surgical interventions include cataract extraction/lensectomy, excisional goniotomy, transscleral cyclophotocoagulation, and glaucoma surgery [5].

This case report aims to demonstrate the challenges in diagnosing and treating bilateral PIS.

## Case presentation

A 49-year-old Malay gentleman with underlying hypertension, subclinical hypothyroidism, and seizure had been diagnosed with bilateral normal-tension glaucoma in March 2023 and was started on topical latanoprost 0.005% once nightly(ON) in both eyes. He was asymptomatic and well during regular follow-up.

In July 2024, he suddenly presented with acute onset of left eye pain and redness and blurring of vision. At presentation, the IOP was 12 mmHg in the right eye (RE) and 52 mmHg in the left eye (LE). Gonioscopy showed grade 0 angles in three quadrants in both eyes (BE). Further examination of the anterior segment showed a shallow anterior chamber with the presence of mild nuclear sclerosis (NS) cataract BE. He was diagnosed with BE PACG and started with four topical antiglaucoma medications over the LE, and subsequently, BE LPI was commenced. The LE IOP decreased but remained elevated, ranging from 20 to 28 mmHg.

During subsequent follow-up, he complained of occipital headache, and IOP was found to have risen again up to 38 mmHg RE and 42 mmHg LE despite patent LPI. He was then admitted to the ward for the proper management of high IOP. 

**Figure 1:**
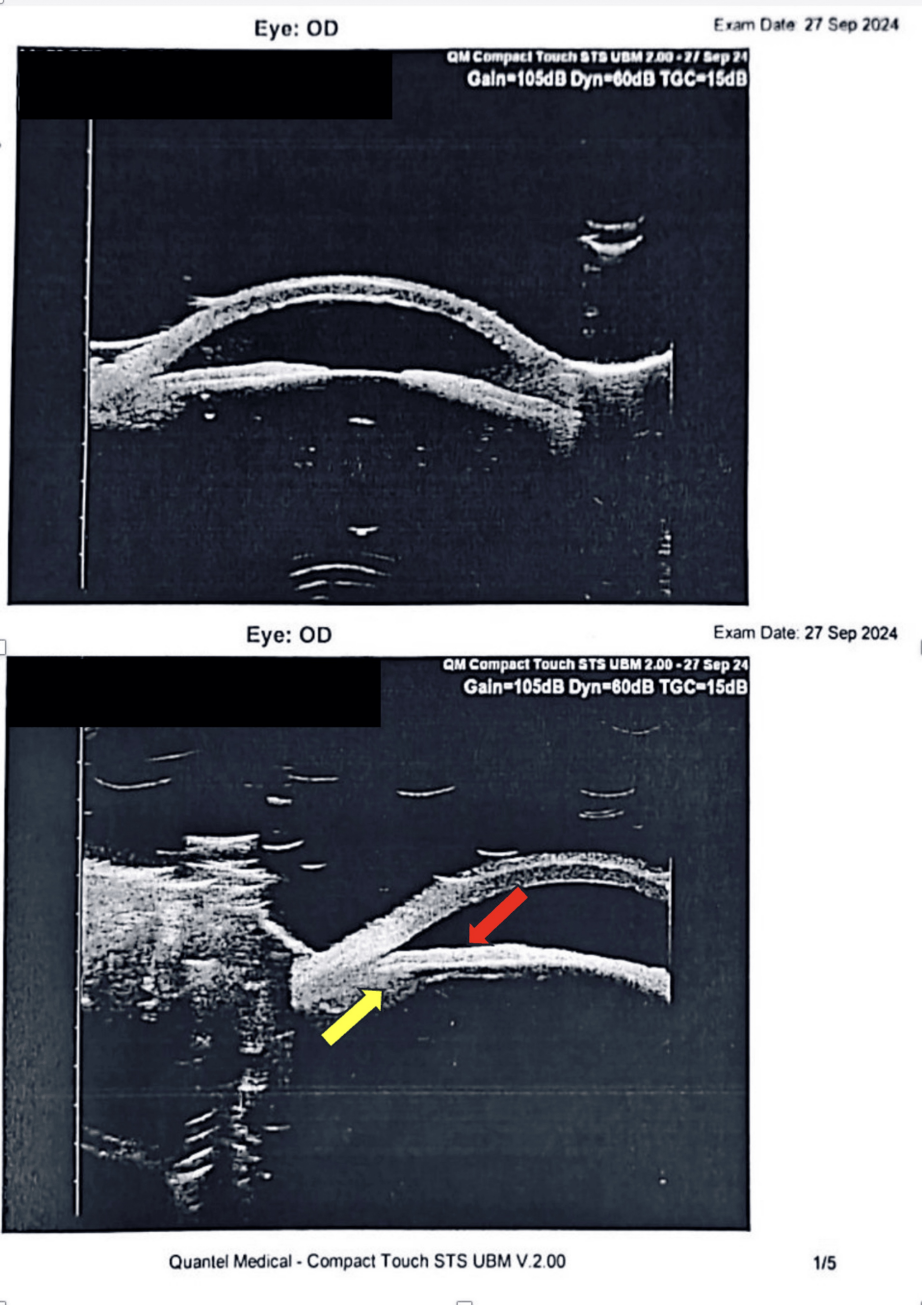
Ultrasound biomicroscopy showing a shallow anterior chamber with anteriorly situated ciliary process (yellow arrow) and feature suggestive of flat iris surface (red arrow)

In the ward, he was on four topical antiglaucoma medications with oral acetazolamide. However, IOP over BE was fluctuating. Repeated gonioscopy showed grade 3 angles in all quadrants in the RE and grade 2-3 angles in the LE with no obvious double-hump sign. UBM demonstrated features suggestive of PIS such as a shallow anterior chamber with a possible anteriorly situated ciliary process and flat iris surface (Figure 1). He was treated with ALPI, and the IOP was reducing in trend; however, it still did not reach the optimum level. He subsequently underwent LE phacoemulsification with intraocular lens (IOL) implantation. IOP of both eyes subsequently was able to reach the optimum level with a reduction in the number of antiglaucoma eyedrops used (Table 1).

**Table 1:** Summary of the initial (pre-treatment) and final (post-treatment) findings of the patient BCVA: best corrected visual acuity; RE: right eye; LE: left eye; BE: both eyes; IOP: intraocular pressure; ON: every night; OM: every morning; TDS: three times daily; CDR: cup-to-disc ratio

Examination findings	Initial	Final (3 months after treatment)
BCVA	RE: 6/7.5; LE: 6/15	RE: 6/6; LE: 6/6
IOP (mmHg)	RE: 12; LE: 52	RE: 12; LE: 14
Optic nerve findings	RE: CDR 0.7, pink; LE: CDR 0.7, pink	RE: CDR 0.7, pink; LE: CDR 0.8, pink
Visual field	RE: no defect; LE: nasal step deformity	RE: nasal step deformity; LE: same, no changes
Medication list	Topical latanoprost ON LE, topical timolol OM LE, topical dorzolamide TDS LE, topical brimonidine TDS LE	Topical timolol OM BE

## Discussion

This case report describes the clinical course of a 49-year-old patient with PIS in whom standard treatment with LPI failed to achieve adequate IOP control, ultimately requiring surgical intervention. At the time of initial NTG diagnosis, gonioscopy findings were not suggestive of angle closure, and the patient had normal IOP with glaucomatous optic disc changes. The later development of angle closure suggests that the patient may have had an underlying PIS that was not clinically evident at initial presentation. The fluctuation in IOP and persistent symptoms post-ALPI highlight the importance of a thorough evaluation and a diverse and complex treatment strategy. The eventual decision to perform phacoemulsification and intraocular lens implantation resulted in excellent results, including optimal IOP control and reduced reliance on topical antiglaucoma medications. This case emphasizes the importance of a thorough follow-up and reassessment after initial therapies, as well as the possibility of surgical intervention when dealing with conditions like PIS that do not respond well to standard treatments.

PIS must be differentiated from other causes of angle-closure glaucoma before a definitive diagnosis can be made. A previous study had highlighted the important distinction between PIS and plateau iris configuration [5]. While both include anterior location of the iris root, PIS lasts even after a patent peripheral iridotomy, as opposed to a plateau iris configuration, which usually disappears. This highlights the need for UBM in accurately assessing anterior segment architecture and confirming diagnoses, as AS-OCT alone may be insufficient to identify plateau iris [10]. In this case, the persistently elevated IOP despite the patent and enlarged PI, together with the UBM findings, significantly supports the diagnosis of PIS. 

A multimodal approach is frequently required for PIS management. While LPI can be effective in some cases, as described in a study by Choy et al., it may not be sufficient for long-term IOP control [11]. Another study reported that although ALPI is highly effective in widening the angle by relieving appositional closure, its effects may diminish over time, and retreatment may be required [6]. In this case, the patient's IOP improved after LPI but remained above the target until phacoemulsification with intraocular lens implantation was performed. This suggests that cataract surgery, when combined with LPI, may play a significant role in PIS management. This finding was consistent with a systematic review demonstrating that phacoemulsification alone can be an effective treatment for PIS, particularly in patients who have failed prior ALPI or are developing visually significant cataracts [12]. In a retrospective review, phacoemulsification resulted in angle opening to Spaeth grades C or D in all treated eyes, with a significant proportion of patients becoming free from IOP-lowering medications while maintaining IOP below 21 mmHg after surgery [9]. The long-term management of PIS is still being researched. Choy et al. reported recurrent angle closure in two patients despite cataract surgery and ALPI, implying that factors such as progressive anterior rotation of ciliary processes and insufficient peripheral treatment may contribute to these issues [11]. For patients presenting with advanced glaucomatous optic neuropathy, extensive peripheral anterior synechiae, or uncontrolled IOP, lens extraction alone may not be sufficient, necessitating the consideration of trabeculectomy or glaucoma drainage device implantation [5]. This emphasizes the importance of ongoing monitoring and individualized treatment strategies for PIS patients. 

This case highlights the diagnostic challenges in PIS, particularly in patients with coexisting cataracts. Management often requires an individualized combination of laser procedures such as LPI or ALPI, and in selected cases, lens extraction to deepen the anterior chamber and improve the angle configuration may be required. While these steps are vital for stabilizing eye pressure and preventing blindness, future research into long-term recovery is recommended to ensure the best strategies and treatments are given for patients facing this complex and unpredictable condition.

## Conclusions

This case report highlights the complexities of diagnosing and managing PIS, especially when it occurs concurrently with cataracts and is initially managed as angle-closure glaucoma, with PIS later recognized as an additional underlying mechanism. The successful management observed after cataract surgery suggests that a multimodal approach, combining LPI and lens extraction, may be required for optimal IOP management. This case adds valuable insights to the existing literature on PIS, emphasizing the importance of increased awareness, advanced diagnostic methodologies, and comprehensive management plans in clinical practice. Additional research is needed to improve our understanding of PIS and optimize patient outcomes in this difficult condition.
